# *catena*-Poly[[bis­(ethanol-κ*O*)mangan­ese(II)]-μ-2,5-di­chloro-3,6-di­oxo­cyclo­hexa-1,4-diene-1,4-bis­(olato)-κ^4^ *O* ^1^,*O* ^6^:*O* ^3^,*O* ^4^]

**DOI:** 10.1107/S1600536814002396

**Published:** 2014-02-08

**Authors:** Seiya Tanaka, Akiko Himegi, Tomomi Ohishi, Akira Fuyuhiro, Satoshi Kawata

**Affiliations:** a Department of Chemistry, Faculty of Science, Fukuoka University, Nanakuma, Jonan-ku, Fukuoka 814-0180, Japan; b Department of Chemistry, Graduate School of Science, Osaka University, Toyonaka, Osaka 560-0043, Japan

## Abstract

In the title coordination polymer, [Mn(C_6_Cl_2_O_4_)(C_2_H_5_OH)_2_]_*n*_, the Mn^II^ atom and the chloranilate [systematic name: 2,5-di­chloro-3,6-dioxo­cyclo­hexa-1,4-diene-1,4-bis­(olate)] ion lie on crystallographic inversion centers. The geometry around the Mn^II^ atom is a distorted octa­hedron involving four O atoms of two chloranilate ions and two O atoms from two ethanol mol­ecules. The chloranilate ion serves as a bridging ligand between the Mn^II^ ions, leading to an infinite linear chain along the *b-*axis direction. The chains are linked by O—H⋯O hydrogen bonds between the apically coordinating ethanol mol­ecule and the chloranilate ion, affording a two-dimensional layer expanding parallel to the *ab* plane.

## Related literature   

For metal complexes of chloranilic acid, see: Kawata *et al.* (1995 ▶, 1998 ▶); Kitagawa *et al.* (1996 ▶); Kitagawa & Kawata (2002 ▶); Abrahams *et al.* (2011 ▶).
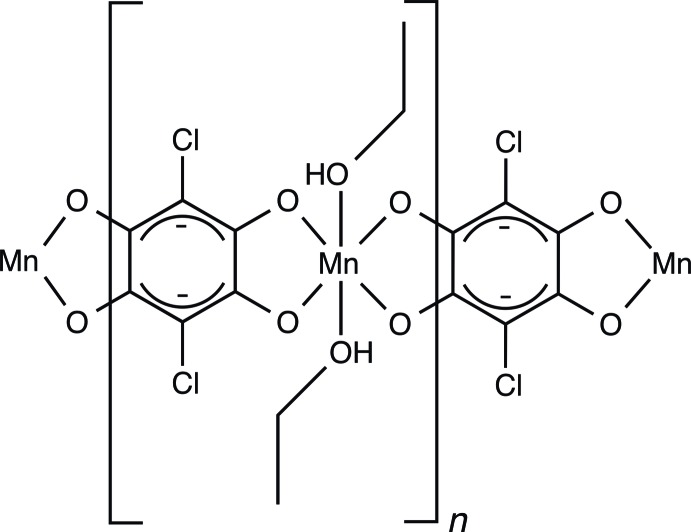


## Experimental   

### 

#### Crystal data  

[Mn(C_6_Cl_2_O_4_)(C_2_H_6_O)_2_]
*M* *_r_* = 354.05
Triclinic, 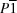

*a* = 5.0784 (5) Å
*b* = 8.1255 (8) Å
*c* = 8.9003 (9) Å
α = 102.718 (4)°
β = 105.175 (5)°
γ = 101.092 (3)°
*V* = 333.35 (6) Å^3^
*Z* = 1
Mo *K*α radiation
μ = 1.41 mm^−1^
*T* = 200 K
0.50 × 0.25 × 0.10 mm

#### Data collection  

Rigaku R-AXIS RAPID II diffractometer
Absorption correction: multi-scan (*ABSCOR*; Rigaku, 1995 ▶) *T*_min_ = 0.406, *T*_max_ = 0.869
3298 measured reflections
1534 independent reflections
1434 reflections with *I* > 2σ(*I*)
*R*_int_ = 0.029

#### Refinement  

*R*[*F* ^2^ > 2σ(*F* ^2^)] = 0.032
*wR*(*F* ^2^) = 0.095
*S* = 1.17
1534 reflections
93 parameters
H atoms treated by a mixture of independent and constrained refinement
Δρ_max_ = 0.70 e Å^−3^
Δρ_min_ = −0.32 e Å^−3^

### 

Data collection: *RAPID-AUTO* (Rigaku, 2002 ▶); cell refinement: *RAPID-AUTO*; data reduction: *RAPID-AUTO*; program(s) used to solve structure: *SIR92* (Altomare *et al.*, 1993 ▶); program(s) used to refine structure: *SHELXL97* (Sheldrick, 2008 ▶); molecular graphics: *CrystalStructure*; software used to prepare material for publication: *CrystalStructure* (Rigaku, 2010 ▶).

## Supplementary Material

Crystal structure: contains datablock(s) General, I. DOI: 10.1107/S1600536814002396/is5335sup1.cif

Structure factors: contains datablock(s) I. DOI: 10.1107/S1600536814002396/is5335Isup2.hkl

CCDC reference:

Additional supporting information:  crystallographic information; 3D view; checkCIF report

## Figures and Tables

**Table 1:** Selected bond lengths (Å)

Mn1—O1	2.1884 (13)
Mn1—O2	2.1491 (11)
Mn1—O3	2.2042 (16)

**Table 2:** Hydrogen-bond geometry (Å, °)

*D*—H⋯*A*	*D*—H	H⋯*A*	*D*⋯*A*	*D*—H⋯*A*
O3—H1⋯O1^i^	0.76 (4)	2.07 (3)	2.8200 (17)	167 (4)

Symmetry code: (i) 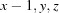.

**Fig. 1.**
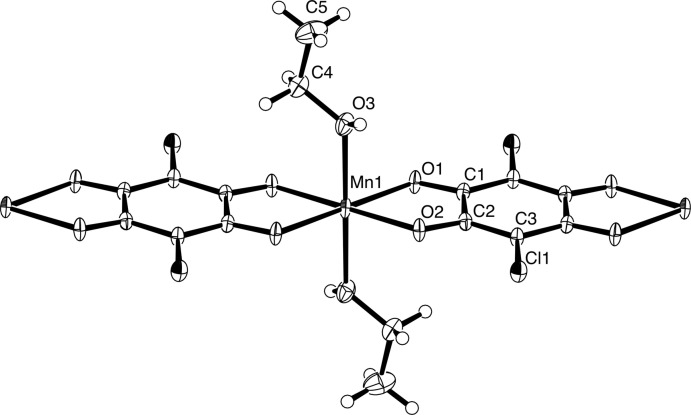
An ORTEP drawing of the title complex, showing 50% probability displacement ellipsoids.

**Fig. 2.**
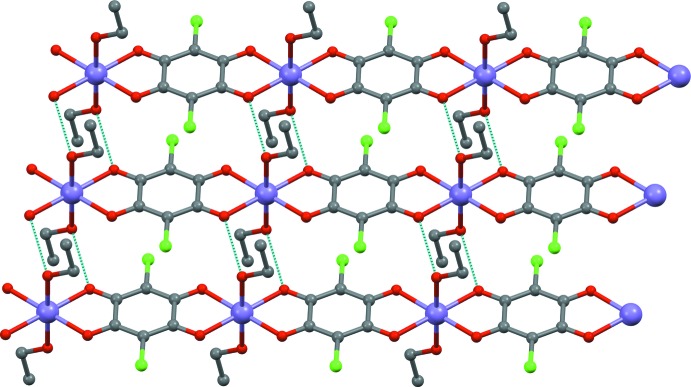
A packing view of the title compound, showing a two-dimensional structure. Blue lines indicate O—H···O hydrogen bonds. H atoms have been omitted for clarity.

## supplementary crystallographic information

### 1. Comment

Benzoquinones and their derivatives have been used and known as bis-bidentate ligands and are good candidates to provide transition metal coordination polymers (Kawata *et al.*, 1995, 1998; Kitagawa *et al.*, 1996; Kitagawa & Kawata, 2002; Abrahams *et al.*, 2011). The background of this chemistry prompts us to utilize chloranilate (CA) chains of Mn as a building block for high dimensional structures. We have succeeded in the synthesis and characterization of a one-dimensional coordination polymer having a hydrogen-bonding link, [Mn(CA)(EtOH)_2_]*_n_* (Fig. 1). The four O atoms of the CA^2-^ anion and the Mn^II^ atom form a basal plane, because the Mn—O distances [2.1884 (13) and 2.1491 (11) Å] are shorter than the two apical Mn—O(EtOH) distances [2.2042 (16) Å]. The hydrogen-bond donor EtOH serves as a woof in the synthesis of a woven polymer: the straight one-dimensional [Mn(CA)(EtOH)_2_]*_n_* chains are linked by two hydrogen bonds [O3—H1···O1 distance: 2.8200 (17) Å] between the apically coordinated EtOH molecule and the O atom of CA^2-^ anion in the nearest neighbor chain to afford a two-dimensional layer (Fig. 2). A similar hydrogen bond is also found between O atoms of water molecules and CA^2-^ anion in [Mn(CA)(H_2_O)_2_(phz)]*_n_* (Kawata *et al.*, 1998), where the straight chains are linked by hydrogen bonds [2.751 (2) Å] shorter than those in the title compound. The inter-chain hydrogen bonds lead to short nearest neighbor Mn···Mn distances [5.6784 (5) Å], and the geometry of the two-dimensional sheet can be regarded as a rectangular array of manganese atoms. The title complex is a good example of lattice structures formed by hydrogen bonds. The fabrication of two-dimensional polymers from warp and woof components has been shown to be quite useful in the construction of tetragonal Mn lattices. This concept can also be applied to a wide variety of compounds having square lattices.

### 2. Experimental

Aqueous solution of MnCl_2_·4H_2_O (5 ml, 30 mmol*L*^-1^) was transferred to a glass tube, and ethanolic solution of H_2_CA (5 ml, 90 mmol*L*^-1^) was poured into the glass tube without mixing the solutions. Green crystals began to form at ambient temperature within one week.

### 3. Refinement

The C-bound H atoms in the ethanol molecule were placed at calculated positions with C—H = 0.98 or 0.99 Å, and were treated as riding on their parent atoms with *U*_iso_(H) set to 1.2*U*_eq_(C). The O-bound H atom in the ethanol molecule was located in a difference Fourier map and refined freely.

### Crystal data

**Table:**

[Mn(C_6_Cl_2_O_4_)(C_2_H_6_O)_2_]	*Z* = 1
*M**_r_* = 354.05	*F*(000) = 179.00
Triclinic, *P*1	*D*_x_ = 1.764 Mg m^−^^3^
Hall symbol: -P 1	Mo *K*α radiation, λ = 0.71075 Å
*a* = 5.0784 (5) Å	Cell parameters from 3040 reflections
*b* = 8.1255 (8) Å	θ = 3.1–27.5°
*c* = 8.9003 (9) Å	µ = 1.41 mm^−^^1^
α = 102.718 (4)°	*T* = 200 K
β = 105.175 (5)°	Block, green
γ = 101.092 (3)°	0.50 × 0.25 × 0.10 mm
*V* = 333.35 (6) Å^3^	

### Data collection

**Table:**

Rigaku R-AXIS RAPID II diffractometer	1434 reflections with *F*^2^ > 2σ(*F*^2^)
Detector resolution: 10.000 pixels mm^-1^	*R*_int_ = 0.029
ω scans	θ_max_ = 27.5°
Absorption correction: multi-scan (*ABSCOR*; Rigaku, 1995)	*h* = −6→6
*T*_min_ = 0.406, *T*_max_ = 0.869	*k* = −10→9
3298 measured reflections	*l* = −11→11
1534 independent reflections	

### Refinement

**Table:**

Refinement on *F*^2^	Secondary atom site location: difference Fourier map
*R*[*F*^2^ > 2σ(*F*^2^)] = 0.032	Hydrogen site location: inferred from neighbouring sites
*wR*(*F*^2^) = 0.095	H atoms treated by a mixture of independent and constrained refinement
*S* = 1.17	*w* = 1/[σ^2^(*F*_o_^2^) + (0.0605*P*)^2^ + 0.0437*P*] where *P* = (*F*_o_^2^ + 2*F*_c_^2^)/3
1534 reflections	(Δ/σ)_max_ < 0.001
93 parameters	Δρ_max_ = 0.70 e Å^−^^3^
0 restraints	Δρ_min_ = −0.32 e Å^−^^3^
Primary atom site location: structure-invariant direct methods	

### Special details

**Table:**

Refinement. Refinement was performed using all reflections. The weighted *R*-factor (*wR*) and goodness of fit (*S*) are based on *F*^2^. *R*-factor (gt) are based on *F*. The threshold expression of *F*^2^ > 2.0 σ(*F*^2^) is used only for calculating *R*-factor (gt).

### Fractional atomic coordinates and isotropic or equivalent isotropic displacement parameters (Å^2^)

**Table:**

	*x*	*y*	*z*	*U*_iso_*/*U*_eq_	
Mn1	1.0000	1.0000	0.0000	0.01772 (15)	
Cl1	0.38362 (8)	0.36866 (5)	−0.26023 (5)	0.02047 (16)	
O1	1.2640 (3)	0.84608 (14)	0.10826 (15)	0.0196 (3)	
O2	0.7400 (3)	0.73489 (15)	−0.09699 (15)	0.0197 (3)	
O3	0.8359 (3)	1.03778 (16)	0.20763 (16)	0.0256 (3)	
C1	1.1523 (3)	0.68293 (19)	0.06530 (18)	0.0154 (3)	
C2	0.8514 (3)	0.6189 (2)	−0.05794 (18)	0.0152 (3)	
C3	0.7193 (4)	0.43901 (19)	−0.11994 (19)	0.0162 (3)	
C4	0.8660 (5)	1.2048 (3)	0.3176 (3)	0.0278 (4)	
C5	0.7319 (5)	1.1860 (4)	0.4470 (3)	0.0426 (6)	
H1	0.691 (7)	0.975 (4)	0.187 (4)	0.049 (8)*	
H4A	0.7772	1.2778	0.2552	0.0334*	
H4B	1.0697	1.2656	0.3696	0.0334*	
H5A	0.5295	1.1279	0.3960	0.0511*	
H5B	0.7570	1.3021	0.5189	0.0511*	
H5C	0.8219	1.1158	0.5104	0.0511*	

### Atomic displacement parameters (Å^2^)

**Table:**

	*U*^11^	*U*^22^	*U*^33^	*U*^12^	*U*^13^	*U*^23^
Mn1	0.0186 (3)	0.0085 (2)	0.0267 (3)	0.00490 (15)	0.00650 (16)	0.00590 (15)
Cl1	0.0161 (3)	0.0159 (3)	0.0247 (3)	0.00325 (16)	0.00050 (17)	0.00448 (17)
O1	0.0177 (6)	0.0086 (5)	0.0296 (7)	0.0025 (5)	0.0034 (5)	0.0055 (5)
O2	0.0183 (6)	0.0107 (6)	0.0296 (7)	0.0049 (5)	0.0041 (5)	0.0078 (5)
O3	0.0236 (7)	0.0177 (6)	0.0340 (7)	0.0020 (6)	0.0125 (6)	0.0037 (5)
C1	0.0157 (8)	0.0110 (7)	0.0204 (8)	0.0039 (6)	0.0071 (6)	0.0043 (6)
C2	0.0149 (7)	0.0128 (7)	0.0204 (8)	0.0051 (6)	0.0069 (6)	0.0065 (6)
C3	0.0148 (7)	0.0109 (7)	0.0215 (8)	0.0035 (6)	0.0033 (6)	0.0049 (6)
C4	0.0275 (10)	0.0221 (9)	0.0308 (10)	0.0060 (7)	0.0092 (8)	0.0022 (7)
C5	0.0331 (11)	0.0515 (14)	0.0345 (12)	0.0023 (10)	0.0145 (9)	−0.0018 (10)

### Geometric parameters (Å, º)

**Table:**

Mn1—O1	2.1884 (13)	C1—C2	1.5410 (19)
Mn1—O1^i^	2.1884 (13)	C1—C3^ii^	1.392 (3)
Mn1—O2	2.1491 (11)	C2—C3	1.402 (2)
Mn1—O2^i^	2.1491 (11)	C4—C5	1.504 (4)
Mn1—O3	2.2042 (16)	O3—H1	0.76 (3)
Mn1—O3^i^	2.2042 (16)	C4—H4A	0.990
Cl1—C3	1.7285 (15)	C4—H4B	0.990
O1—C1	1.2646 (18)	C5—H5A	0.980
O2—C2	1.255 (3)	C5—H5B	0.980
O3—C4	1.442 (3)	C5—H5C	0.980
			
O1—Mn1—O1^i^	180.00 (7)	O2—C2—C1	116.48 (13)
O1—Mn1—O2	75.40 (5)	O2—C2—C3	124.00 (13)
O1—Mn1—O2^i^	104.60 (5)	C1—C2—C3	119.52 (15)
O1—Mn1—O3	89.94 (6)	Cl1—C3—C1^ii^	119.54 (10)
O1—Mn1—O3^i^	90.06 (6)	Cl1—C3—C2	119.10 (13)
O1^i^—Mn1—O2	104.60 (5)	C1^ii^—C3—C2	121.29 (13)
O1^i^—Mn1—O2^i^	75.40 (5)	O3—C4—C5	112.07 (17)
O1^i^—Mn1—O3	90.06 (6)	Mn1—O3—H1	112 (3)
O1^i^—Mn1—O3^i^	89.94 (6)	C4—O3—H1	112 (3)
O2—Mn1—O2^i^	180.00 (8)	O3—C4—H4A	109.195
O2—Mn1—O3	90.47 (5)	O3—C4—H4B	109.194
O2—Mn1—O3^i^	89.53 (5)	C5—C4—H4A	109.198
O2^i^—Mn1—O3	89.53 (5)	C5—C4—H4B	109.199
O2^i^—Mn1—O3^i^	90.47 (5)	H4A—C4—H4B	107.893
O3—Mn1—O3^i^	180.00 (7)	C4—C5—H5A	109.468
Mn1—O1—C1	115.42 (10)	C4—C5—H5B	109.470
Mn1—O2—C2	116.70 (9)	C4—C5—H5C	109.467
Mn1—O3—C4	125.08 (13)	H5A—C5—H5B	109.476
O1—C1—C2	115.83 (15)	H5A—C5—H5C	109.471
O1—C1—C3^ii^	125.09 (13)	H5B—C5—H5C	109.475
C2—C1—C3^ii^	119.07 (13)		
			
O1—Mn1—O2—C2	−3.21 (9)	O3—Mn1—O2^i^—C2^i^	−86.98 (10)
O2—Mn1—O1—C1	0.99 (9)	O2^i^—Mn1—O3^i^—C4^i^	165.27 (10)
O1—Mn1—O2^i^—C2^i^	−176.79 (9)	O3^i^—Mn1—O2^i^—C2^i^	93.02 (10)
O2^i^—Mn1—O1—C1	−179.01 (9)	Mn1—O1—C1—C2	0.92 (19)
O1—Mn1—O3—C4	119.34 (10)	Mn1—O1—C1—C3^ii^	−179.43 (11)
O3—Mn1—O1—C1	91.49 (10)	Mn1—O2—C2—C1	4.67 (19)
O1—Mn1—O3^i^—C4^i^	60.66 (10)	Mn1—O2—C2—C3	−175.03 (11)
O3^i^—Mn1—O1—C1	−88.51 (10)	Mn1—O3—C4—C5	−179.70 (9)
O1^i^—Mn1—O2—C2	176.79 (9)	O1—C1—C2—O2	−3.8 (3)
O2—Mn1—O1^i^—C1^i^	179.01 (9)	O1—C1—C2—C3	175.92 (15)
O1^i^—Mn1—O2^i^—C2^i^	3.21 (9)	O1—C1—C3^ii^—Cl1^ii^	1.2 (3)
O2^i^—Mn1—O1^i^—C1^i^	−0.99 (9)	O1—C1—C3^ii^—C2^ii^	−175.82 (16)
O1^i^—Mn1—O3—C4	−60.66 (10)	C2—C1—C3^ii^—Cl1^ii^	−179.14 (13)
O3—Mn1—O1^i^—C1^i^	88.51 (10)	C2—C1—C3^ii^—C2^ii^	3.8 (3)
O1^i^—Mn1—O3^i^—C4^i^	−119.34 (10)	C3^ii^—C1—C2—O2	176.54 (15)
O3^i^—Mn1—O1^i^—C1^i^	−91.49 (10)	C3^ii^—C1—C2—C3	−3.7 (3)
O2—Mn1—O3—C4	−165.27 (10)	O2—C2—C3—Cl1	0.6 (3)
O3—Mn1—O2—C2	−93.02 (10)	O2—C2—C3—C1^ii^	−176.48 (16)
O2—Mn1—O3^i^—C4^i^	−14.73 (10)	C1—C2—C3—Cl1	−179.11 (13)
O3^i^—Mn1—O2—C2	86.98 (10)	C1—C2—C3—C1^ii^	3.8 (3)
O2^i^—Mn1—O3—C4	14.73 (10)		

Symmetry codes: (i) −*x*+2, −*y*+2, −*z*; (ii) −*x*+2, −*y*+1, −*z*.

### Hydrogen-bond geometry (Å, º)

**Table:**

*D*—H···*A*	*D*—H	H···*A*	*D*···*A*	*D*—H···*A*
O3—H1···O1^iii^	0.76 (4)	2.07 (3)	2.8200 (17)	167 (4)

Symmetry code: (iii) *x*−1, *y*, *z*.
